# Laparoscopic total colectomy in a patient with familial adenomatous polyposis and congenital intestinal nonrotation: a case report

**DOI:** 10.1093/jscr/rjag711

**Published:** 2026-08-23

**Authors:** Kavya Jasti, Wilder R Calmet Rocca, Ashley Hopfinger, Walter A Ramsey, Daniela Rebollo, Shayan Khalafi, David D Zhang, Laurence R Sands, Vanessa Hui, Nivedh V Paluvoi, Austin R Dosch

**Affiliations:** University of Miami Miller School of Medicine, 1600 NW 10th Avenue, Suite 1149 Miami, FL 33136, United States; Facultad De Medicina Alberto Hurtado, Universidad Peruana Cayetano Heredia, Av. Honorio Delgado 430, Urb. Ingeniería, San Martín de Porres, Lima 15102, Peru; DeWitt Daughtry Family Department of Surgery, University of Miami Miller School of Medicine, 1600 NW 10th Ave, Miami, FL 33136, United States; DeWitt Daughtry Family Department of Surgery, University of Miami Miller School of Medicine, 1600 NW 10th Ave, Miami, FL 33136, United States; DeWitt Daughtry Family Department of Surgery, University of Miami Miller School of Medicine, 1600 NW 10th Ave, Miami, FL 33136, United States; DeWitt Daughtry Family Department of Surgery, University of Miami Miller School of Medicine, 1600 NW 10th Ave, Miami, FL 33136, United States; DeWitt Daughtry Family Department of Surgery, University of Miami Miller School of Medicine, 1600 NW 10th Ave, Miami, FL 33136, United States; DeWitt Daughtry Family Department of Surgery, University of Miami Miller School of Medicine, 1600 NW 10th Ave, Miami, FL 33136, United States; DeWitt Daughtry Family Department of Surgery, University of Miami Miller School of Medicine, 1600 NW 10th Ave, Miami, FL 33136, United States; DeWitt Daughtry Family Department of Surgery, University of Miami Miller School of Medicine, 1600 NW 10th Ave, Miami, FL 33136, United States; DeWitt Daughtry Family Department of Surgery, University of Miami Miller School of Medicine, 1600 NW 10th Ave, Miami, FL 33136, United States

**Keywords:** congenital intestinal nonrotation, familial adenomatous polyposis, laparoscopic colectomy, ileorectal anastomosis, case report

## Abstract

Intestinal nonrotation is a rare congenital anomaly resulting from incomplete midgut rotation during embryogenesis. Adult cases represent only 0.2% of the incidence of the disease. Familial adenomatous polyposis (FAP) is an inherited condition characterized by hundreds of colorectal adenomas and a near-100% lifetime risk of colorectal cancer. The coexistence of these two conditions has not been previously reported. A 28-year-old male presented with rectal bleeding. Laboratory evaluation revealed severe anemia and thrombocytopenia. Computed tomography revealed incidental intestinal nonrotation, with small bowel on the right and colon on the left, along with inversion of the superior mesenteric artery and vein. Sigmoidoscopy showed extensive polyposis consistent with FAP. The patient underwent laparoscopic total colectomy with ileorectal anastomosis. This case underscores the importance of identifying congenital anomalies such as nonrotation, and also reinforces the preoperative imaging as critical in anticipating anatomical variations, enabling careful surgical planning, and vessel preservation.

## Introduction

Congenital intestinal nonrotation is a subtype of malrotation secondary to intestinal rotation failure, resulting in the colon on the left side of the abdomen and the small bowel on the right. While 90% of patients present in infancy with signs of volvulus, presentation in adults is often asymptomatic and rare with an incidence of 0.2% [1, 2].

Familial adenomatous polyposis (FAP) is an autosomal dominant condition caused by adenomatous polyposis coli mutation and characterized by extensive colorectal adenomas, predisposing individuals to colorectal cancer with a lifetime risk of almost 100% without prophylactic colectomy or proctocolectomy [3–5].

We present a case of symptomatic FAP with incidental intestinal nonrotation who underwent laparoscopic total colectomy, representing a rare coexistence of these conditions.

## Case report

This is a 28-year-old male without any relevant medical history or family history who presented to the emergency department with rectal bleeding for the past few months. He had no other associated symptoms. Initial laboratory workup found a hemoglobin of 0.0033 g/dL and platelets of 17k.

**Figure 1:**
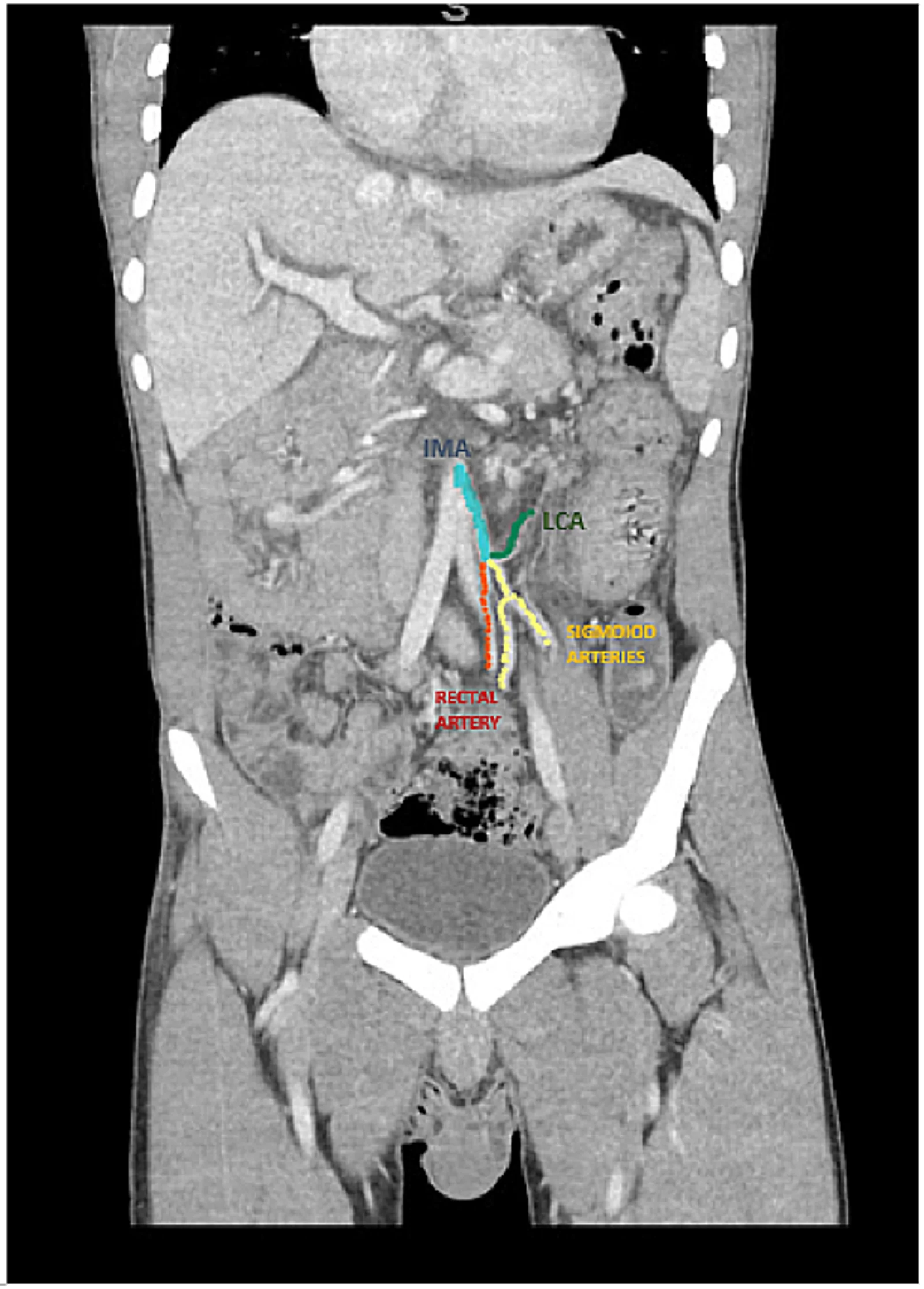
CTA of the abdomen/pelvis showing the IMA, left colic artery, rectal artery, and sigmoid arteries.

**Figure 2:**
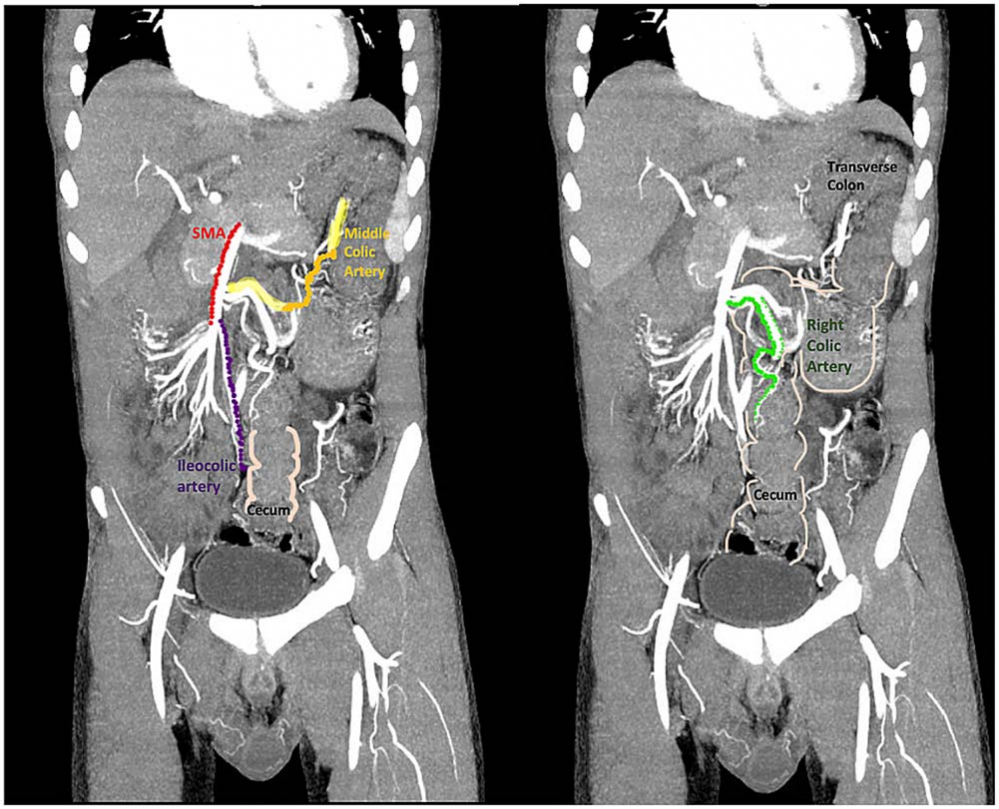
CTA of the abdomen/pelvis with visualization of the SMA and its branches.

Computed tomography angiography (CTA) of the abdomen and pelvis revealed intestinal nonrotation with left-sided colon, right-sided small bowel, and inversion of the superior mesenteric artery (SMA)/superior mesenteric vein (SMV) relationship (Figs 1 and 2).

A flexible sigmoidoscopy was performed for evaluation of rectal bleeding. Diffuse polyposis beginning at the rectosigmoid junction was found, which was concerning for FAP. There was also a rectal polyp burden, which was deemed to be endoscopically clearable. After discussion with the patient regarding strict endoscopic surveillance versus surgical intervention, the patient was scheduled for laparoscopic total colectomy with ileorectal anastomosis.

**Figure 3:**
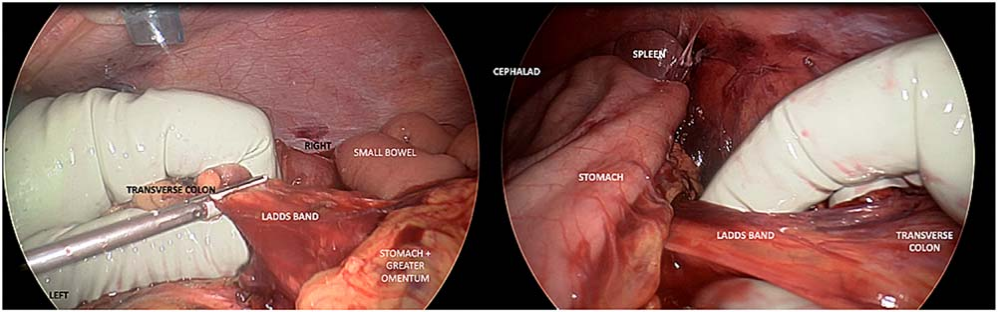
Ladd’s bands tethering the transverse colon to the liver, duodenum, gallbladder, and stomach.

Upon entering the abdomen, the entire small intestine was found in the right hemiabdomen, and the cecum was seen laying over the sacral promontory. The left colon was in the correct anatomical position with an intact white line of Toldt. The right colon was tethered to the small bowel mesentery and retroperitoneum rather than the right abdominal wall. Ladd’s bands connecting the transverse colon to the liver, duodenum, gallbladder, and stomach were also observed (Fig. 3).

**Figure 4:**
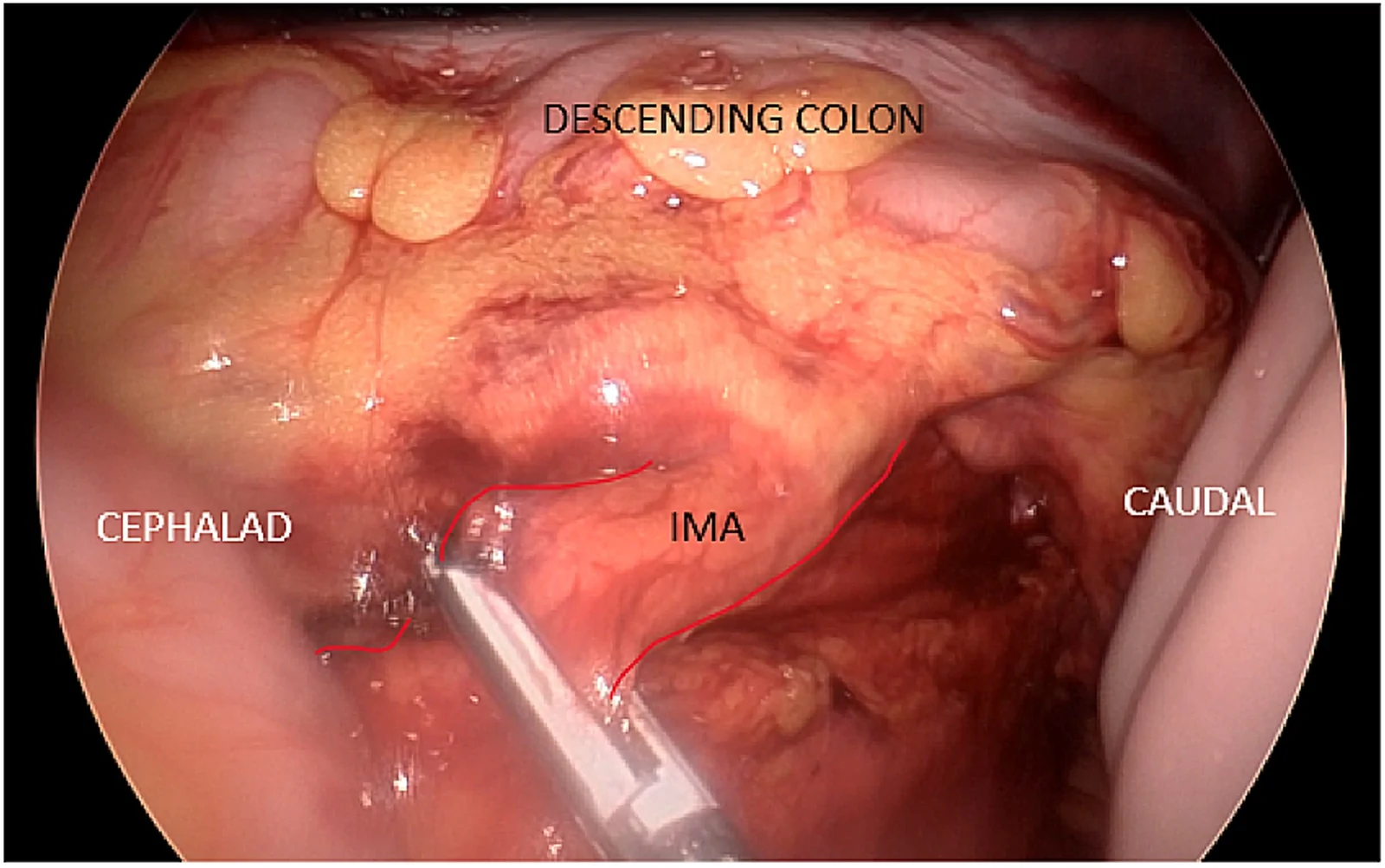
High ligation of the IMA.

The transverse and left colon were mobilized first via lysis of Ladd’s bands. High ligation of the inferior mesenteric artery (IMA) was performed (Fig. 4). The mesentery to the origin of the middle colic vessels was then divided. The middle colon trunk was divided using the Ligasure device. Due to the abnormal anatomy, the inferior mesenteric vein was not visualized.

**Figure 5:**
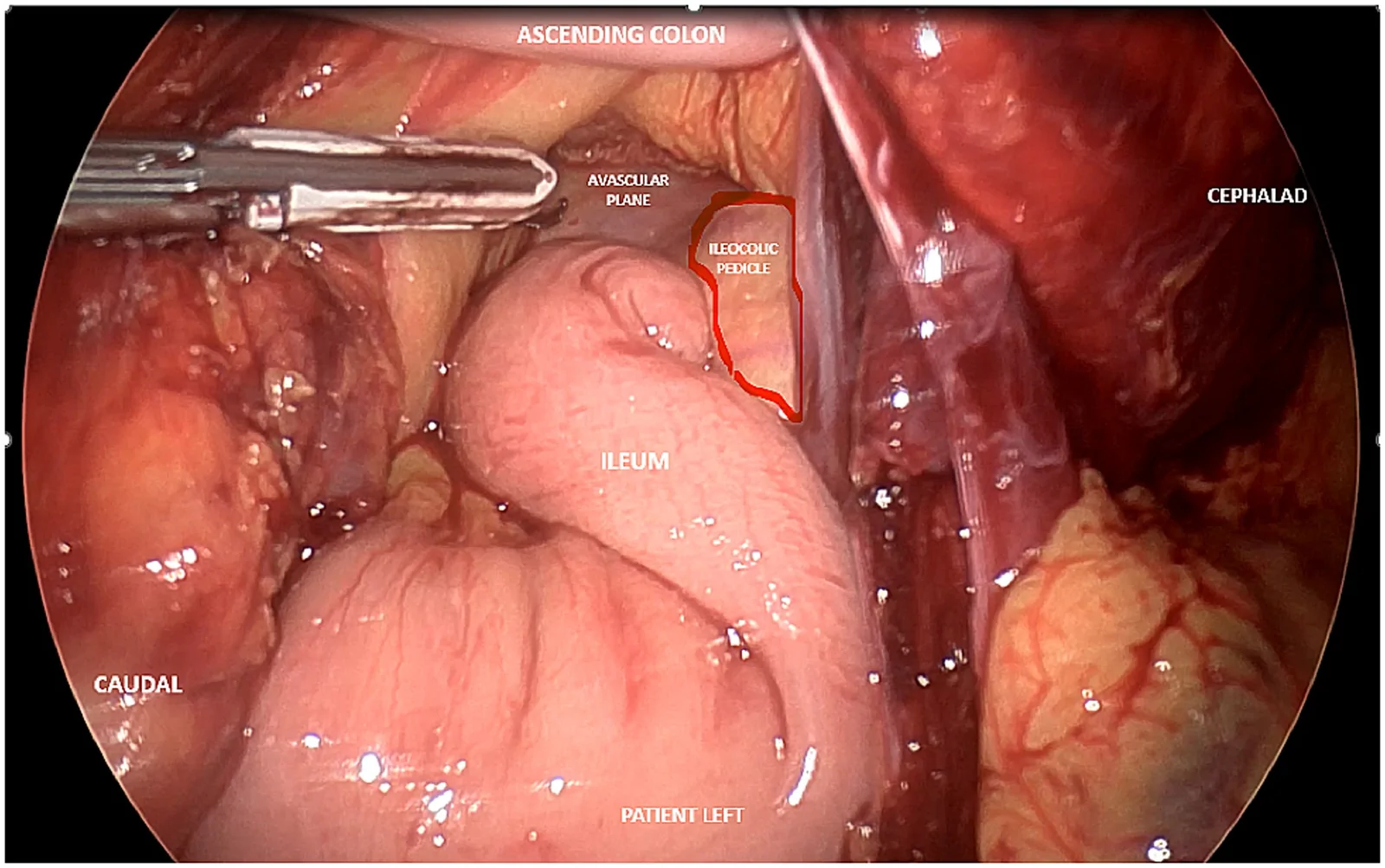
Ileocolic pedicle.

**Figure 6:**
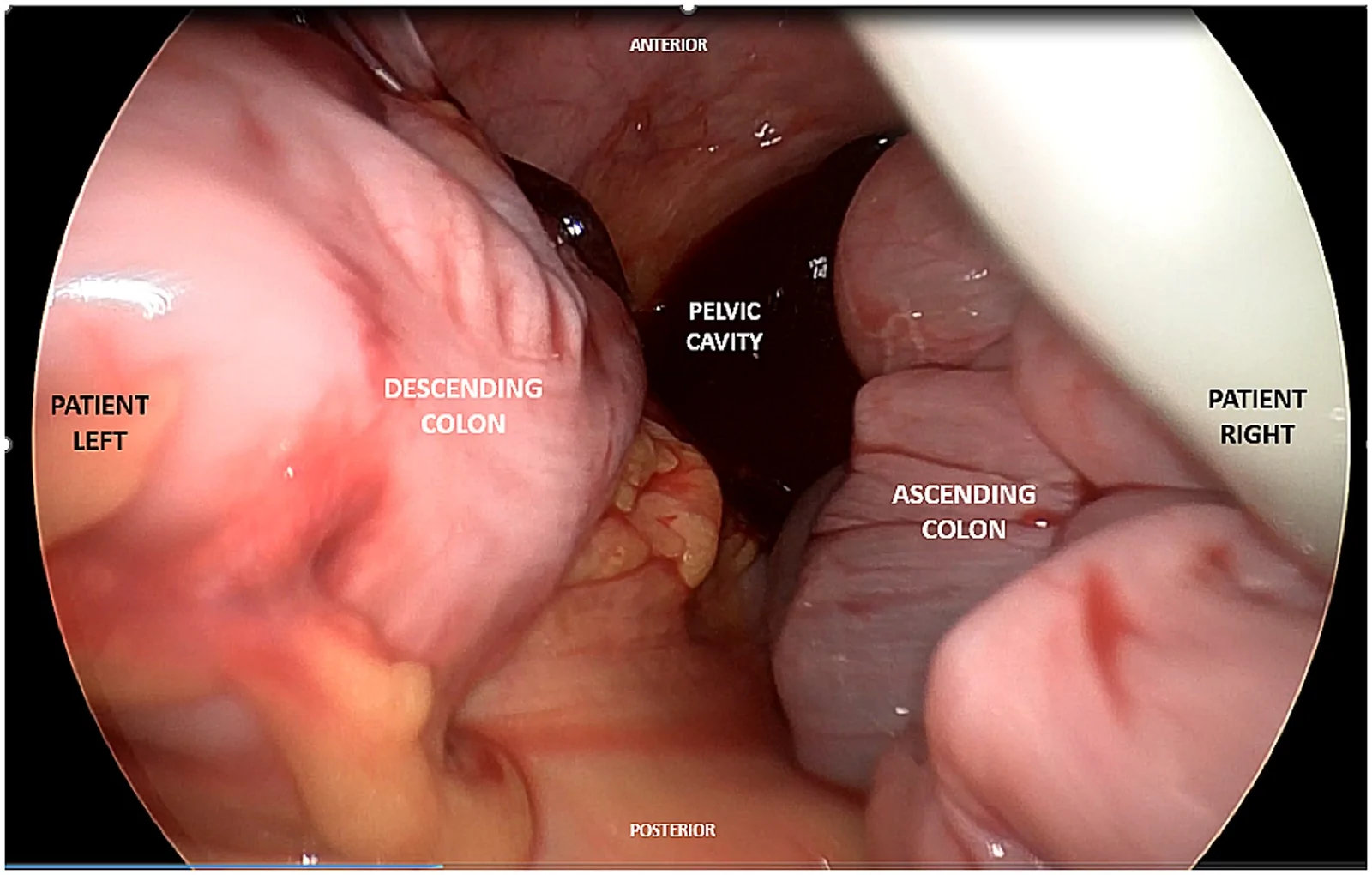
Avascular plane behind the ileocolic pedicle.

The right colon was mobilized next. The ileocolic pedicle was identified, and the avascular plane underneath the vessel was entered, at which the SMV was seen inferiorly and preserved (Figs 5 and 6). High ligation of the ileocolic pedicle was performed. A pulse in the SMA and throughout the small intestine was confirmed. The terminal ileum was divided, and the upper rectum was mobilized. The ileorectal anastomosis was constructed using an end-to-end anastomosis stapling device via the rectum. Flexible endoscopy showed a healthy anastomosis with a negative leak test. The small bowel was evaluated after and appeared well perfused.

The patient’s postoperative recovery was complicated by an ileus and persistent thrombocytopenia**,** which was determined to be secondary to immune thrombocytopenia. The patient was discharged on postoperative day 12.

Pathology of the colon specimen found more than 100 tubular adenomas, some with foci of high-grade dysplasia. The distal colon margin was positive for low-grade dysplasia. The ileum was negative for dysplasia. The specimen overall was negative for carcinoma.

## Discussion

Intestinal malrotation is a congenital anomaly which is predominantly diagnosed within the first year of life. Adults and adolescents with this condition represent only 0.2%–0.5% of the cases [6]. Most patients are asymptomatic, with an incidental discovery of this condition during surgical intervention or pre-operative workup as in our case [7].

**Figure 7:**
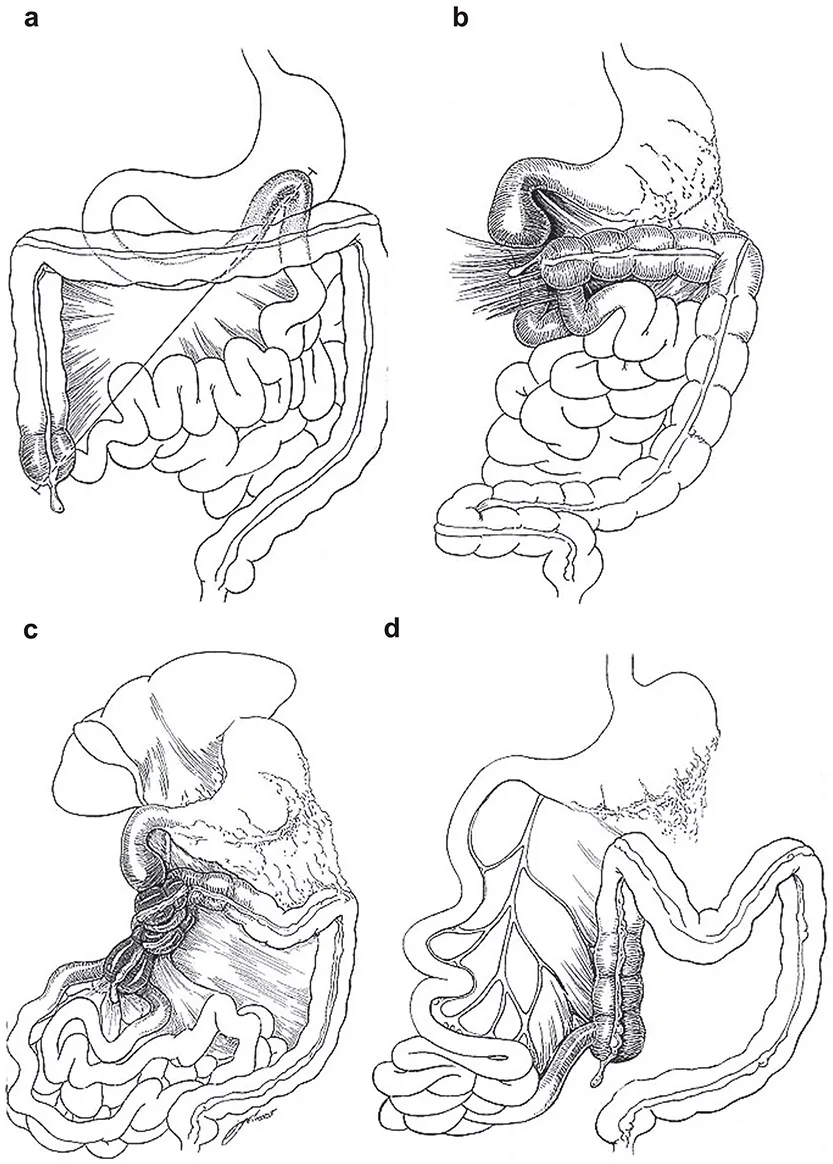
(a) Normal intestinal rotation, (b) malrotation without volvulus, (c) malrotation with volvulus, (d) nonrotation [8].

Normal midgut rotation involves a 270° counterclockwise rotation around the SMA [8] (Fig. 7a). In nonrotation, this process is incomplete, resulting in a left-sided colon and right-sided small bowel (Fig. 7d). Unlike classic malrotation, nonrotation typically results in a wide mesenteric base, which reduces the risk of volvulus. Consequently, many individuals remain asymptomatic and the anomaly is discovered incidentally, as in this patient [8].

In surgical cases involving major abdominal surgery with incidental nonrotation, preoperative imaging is crucial in identifying the abnormal orientation of vasculature. Ultrasounds, computed tomography scans, magnetic resonance imaging, and even mesenteric arteriography can be used to diagnose midgut malrotation [9, 10]. The preoperative CTA revealed inversion of the SMA and SMV as well as the abnormal location of the small and large intestines which allowed our surgical team to anticipate the abnormal vasculature and structures and perform careful dissection for vessel preservation.

FAP is a high penetrance autosomal dominant disease characterized by development of colorectal adenomas that starts in childhood and will progress inevitably to colorectal cancer by the fourth or fifth decade of life [11]. In patients with FAP, the surgical options for the patients include colectomy with ileorectal anastomosis or proctocolectomy with either permanent ileostomy or ileal pouch-anal anastomosis [12]. This is determined by factors such as age, sex, family history, acceptance of a stoma, the rectal polyp burden, concern for infertility and sexual dysfunction, ability to follow-up with endoscopic surveillance, and many other factors [13]. Ileorectal anastomosis was chosen for this patient due to his young age, male sex, and low rectal polyp burden (<20 polyps), thus allowing for preserved rectal function.

This case emphasizes the importance of recognizing congenital anomalies that alter normal anatomy during preoperative assessment. Imaging is crucial in informing the surgical team of variations in vasculature location, thus preventing intraoperative complications.

In conclusion, this case underscores the importance of recognizing incidental intestinal nonrotation when planning major abdominal surgery. Although largely asymptomatic, nonrotation can cause significant changes in the location of vasculature and structures, in turn leading to intraoperative complications if such anomalies are not anticipated. This rare coexistence of FAP and nonrotation highlights the need for individualized surgical planning.

## Consent

Written informed consent for the publication of this case report and accompanying images was obtained from the patient. A copy of the consent is available for review by the Editor-in-Chief of this journal upon request.
